# A Deep Learning-Based Method for Mechanical Equipment Unknown Fault Detection in the Industrial Internet of Things

**DOI:** 10.3390/s25195984

**Published:** 2025-09-27

**Authors:** Xiaokai Liu, Xiangheng Meng, Lina Ning, Fangmin Xu, Qiguang Li, Chenglin Zhao

**Affiliations:** 1 School of Mechanical and Electrical Engineering, Beijing Information Science and Technology University, Beijing 102206, China; liu_xiaokai@bistu.edu.cn (X.L.); lqg0920@bistu.edu.cn (Q.L.); 2 School of Information and Communication Engineering, Beijing University of Posts and Telecommunications, Beijing 100876, China; mengxh@bupt.edu.cn (X.M.); clzhao@bupt.edu.cn (C.Z.); 3 Academy of Military Sciences, Beijing 100097, China

**Keywords:** industrial internet of things, fault diagnosis, autoencoder network, convolutional neural network, long short-term memory network

## Abstract

With the development of the Industrial Internet of Things (IIoT) technology, fault diagnosis has emerged as a critical component of its operational reliability, and machine learning algorithms play a crucial role in fault diagnosis. To achieve better fault diagnosis results, it is necessary to have a sufficient number of fault samples participating in the training of the model. In actual industrial scenarios, it is often difficult to obtain fault samples, and there may even be situations where no fault samples exist. For scenarios without fault samples, accurately identifying the unknown faults of equipment is an issue that requires focused attention. This paper presents a method for the normal-sample-based mechanical equipment unknown fault detection. By leveraging the characteristics of the autoencoder network (AE) in deep learning for feature extraction and sample reconstruction, normal samples are used to train the AE network. Whether the input sample is abnormal is determined via the reconstruction error and a threshold value, achieving the goal of anomaly detection without relying on fault samples. In terms of input data, the frequency domain features of normal samples are used to train the AE network, which improves the training stability of the AE network model, reduces the network parameters, and saves the occupied memory space at the same time. Moreover, this paper further improves the network based on the traditional AE network by incorporating a convolutional neural network (CNN) and a long short-term memory network (LSTM). This enhances the ability of the AE network to extract the spatial and temporal features of the input data, further improving the network’s ability to extract and recognize abnormal features. In the simulation part, through public datasets collected in factories, the advantages and practicality of this method compared with other algorithms in the detection of unknown faults are fully verified.

## 1. Introduction

With the rapid technological evolutions in manufacturing, we are now experiencing a new generation of industrial revolution, that is IIoT [1,2,3,4,5]. In critical sectors covered by the Industrial Internet, such as manufacturing, energy, transportation, and healthcare, equipment failures can lead to production line disruptions, inefficient resource utilization, safety risks, and ecological and environmental hazards [6,7,8]. Typical scenarios include the following: downtime of key processing equipment in discrete manufacturing workshops, which can incur direct production losses ranging from hundreds to tens of thousands of dollars per hour for a single machine; failures in energy transmission and distribution systems, which may cause regional power outages affecting tens of thousands of users; and malfunctions of core components in rail transit, which could jeopardize operational safety. Therefore, establishing a precise equipment fault diagnosis system to achieve early defect identification and potential fault prediction has become a core technical requirement for safeguarding industrial systems’ reliability, safety, and economic efficiency.

However, traditional supervised learning methods depend heavily on abundant labeled fault samples, which are rarely available in industrial environments due to the randomness of failures and the preventive replacement of key components [9,10]. This scarcity necessitates developing detection methods capable of identifying unknown faults under few- or zero-fault-sample conditions [11,12,13,14]. Manual inspections and fixed-interval maintenance are inefficient, lack real-time degradation monitoring, and cannot reliably predict sudden failures, highlighting the urgent need for intelligent predictive maintenance [15]. In the field of industrial Internet equipment fault diagnosis, deep learning technologies, by constructing multi-layer nonlinear mapping networks, have provided a revolutionary solution for unknown fault detection. As a typical unsupervised learning paradigm, the autoencoder (AE) minimizes the mean squared error between the input and the reconstructed output, realizing feature compression and reconstruction of high-dimensional signals in the hidden layer [16]. Its core advantage lies in the fact that it only requires normal samples for training and detects anomalies by identifying reconstruction errors that exceed an adaptive threshold [17]. Traditional AEs and their variants (such as sparse autoencoders and deep convolutional autoencoders) perform excellently in static data processing. However, when facing dynamic signals such as time-series vibrations and currents collected by industrial sensors, due to the lack of the ability to model the correlation of time series and spatial local features, the accuracy of abnormal identification is limited [18]. To address this, we integrate CNN for spatial feature extraction and LSTM for temporal modeling, enhancing fault representation in complex conditions [19]. In industrial predictive maintenance, deep learning-based fault detection offers distinct advantages: by continuously collecting operational data, the model can track reconstruction error changes in real time to identify early-stage performance degradation. Once an anomaly is detected and addressed, the newly acquired fault data can be used for model updates, forming a closed-loop process of “detection–maintenance–optimization” [20]. This capability enables the system not only to detect unknown faults but also to accurately predict the types of faults through the continuous learning of historical fault patterns, guide the formulation of targeted maintenance strategies, greatly improve the operation and maintenance efficiency and reliability of industrial equipment, and promote the paradigm shift of smart factories from passive maintenance to active health management [21].

Amid substantial research on fault detection, deep learning has become a pivotal direction in fault detection due to its ability to automatically extract representative features and handle complex nonlinear data with strong adaptability and generalization [22]. An intelligent diagnostic method utilizing Deep Neural Networks (DNNs) was proposed in [23] to address the shortcomings of traditional methods in handling complex nonlinear data. With the prominence of CNN’s capability in local feature extraction, the authors in the paper [24] proposed a CNN-based prediction model, which achieved the classification of mechanical equipment faults and attained high accuracy even with limited data sources. To further improve the performance of algorithms in equipment fault diagnosis, researchers have begun to combine neural network algorithms with traditional machine learning algorithms. In [25], the researchers integrated CNN with SVM to propose a CNN-SVM bearing fault diagnosis scheme, which uses CNN to extract data features and then employs SVM for classification. This approach reduces system runtime while improving classification accuracy. In reference [26], the authors developed a novel RF-based CNN model, incorporating a dropout layer into the CNN to prevent overfitting. Experimental results confirmed that this method outperforms traditional algorithms such as SVM, CNN, and RF. As input time-series data lengthen, Long Short-Term Memory (LSTM) networks have been adopted in fault diagnosis to capture long-range temporal dependencies. Reference [27] presents a fault diagnosis method for wind turbine gearboxes leveraging an LSTM-based approach. It optimizes the network with cosine loss to reduce the impact of signal intensity on diagnostic accuracy and enhance diagnostic precision. The integration of CNN and LSTM enables simultaneous extraction of spatial and temporal features for fault diagnosis. For example, Reference [28] developed an end-to-end CNN–LSTM model using raw sensor data, and Reference [29] introduced a bidirectional convolutional LSTM to address complex planetary gearbox responses. In reference [30], the authors explored generative adversarial networks combined with deep extreme learning machines to enhance fault diagnosis performance under data-scarce conditions. Reference [31] enhanced detection accuracy by incorporating raw, Fourier-, and wavelet-transformed signals into a multichannel convolutional LSTM, maintaining high performance even with short input segments. Recent studies have increasingly applied Transformer architectures to fault detection and diagnosis tasks in industrial systems. Wu et al. [32] developed a Transformer-based approach for rotary machinery that can classify known faults and detect novel fault types. Zhang et al. [33] proposed TSViT, which leverages a time-series vision Transformer to improve diagnostic performance on rotating machinery datasets. More recently, Xiao et al. [34] introduced a Bayesian variational Transformer that enhances robustness under small-sample and noisy conditions. These works collectively indicate that Transformer-based models are becoming strong baselines in intelligent fault diagnosis. The papers [35,36,37,38,39,40,41] report contributions to the anomaly detection of equipment from different perspectives. The development of autoencoder (AE) networks in the field of deep learning has provided new insights for anomaly detection in mechanical equipment. AE is an unsupervised neural network model with a symmetric network structure [42]. When trained only on normal samples, an autoencoder learns to accurately reconstruct them, yielding low reconstruction errors. In contrast, abnormal samples—unseen during training—are poorly reconstructed, producing higher errors. By setting a reasonable reconstruction error threshold, abnormal samples can be accurately detected [43]. While AEs trained on normal samples are effective for anomaly detection [44,45,46,47,48,49], they are unable to recognize unseen fault types, underscoring the need for methods dedicated to unknown fault detection. Table 1 presents a comparative summary of the proposed DC-LSTM-AE model and related deep learning methods cited in this study.

In summary, autoencoders (AEs) trained exclusively on normal samples are effective at learning representations of normal conditions, but they inherently lack the ability to recognize or categorize abnormal samples. This limitation highlights a fundamental drawback of existing industrial fault detection methods. Although models trained on normal data or predefined fault classes can achieve good performance within their training scope, they are unable to identify previously unseen fault types. This shortcoming poses significant risks in practical applications, where unexpected faults may occur due to the stochastic nature of equipment degradation and operational variability, potentially leading to misjudgments by maintenance personnel, reduced diagnostic efficiency, and avoidable production losses. Accordingly, the objective of this study is to explore whether deep neural networks, trained exclusively on normal operating data, can effectively identify unknown faults in industrial equipment without requiring prior fault samples. To explore this question, we propose a novel deep learning-based method for unknown fault detection that relies exclusively on normal samples during training, enabling the identification of unseen fault types without prior labeling. This paper presents the following primary contributions to fault detection in IIoT:

1. This paper proposes a DC-LSTM-AE model based on deep CNN and LSTM. The model extracts spatial features via a five-layer CNN and captures the long-term dependencies of time-series data through LSTM, enabling spatiotemporal feature fusion for high-dimensional nonlinear time-series signals in industrial environments. This approach addresses traditional autoencoders’ limitations in extracting features from complex signals, enabling accurate reconstruction-based identification of unknown faults without requiring labeled fault samples.
2. To address the core problem of scarce fault samples in industrial scenarios, we design a training procedure using only normal samples. By leveraging the reconstruction error characteristics of autoencoders, a benchmark feature space is constructed through training with normal samples. When abnormal samples are input, their absence from the training process leads to significantly increased reconstruction errors. Anomaly detection is achieved by setting thresholds based on the Pauta criterion. This strategy breaks through the dependence of traditional supervised learning on fault samples, providing a feasible solution for early equipment maintenance.
3. In this study, we employ sliding windows and fast Fourier transform (FFT) to convert time-series signals into spectral features. This reduces data dimensions while preserving key information, enhances model training stability, and lowers memory consumption. The L2 regularization term is introduced to optimize the loss function, suppress overfitting, and enhance the model’s generalization ability. By dynamically adjusting the regularization coefficient through cross-validation, a balance is achieved between model complexity and detection accuracy, making it suitable for real-time detection requirements in industrial sites.
4. We evaluate our method on two industrial datasets: the Southeast University gearbox dataset (four fault types) and a constant-speed water pump dataset from factory settings (one fault type). Compared with traditional autoencoders, Deep Convolutional Autoencoders (DCAEs), and several machine learning algorithms, DC-LSTM-AE achieves notably higher accuracy and precision. In particular, it better distinguishes reconstruction errors for unknown faults with high feature similarity, confirming its effectiveness and industrial applicability.

These contributions collectively advance the understanding and development of fault detection problems in IIoT, providing a robust foundation for future work and practical applications. The remainder of this article is organized as follows: Section 2 provides the system models and an overview of the fault detection model in the IIoT situation. The fault detection strategy based on the DC-LSTM-AE algorithm is created in Section 3. The simulation results and performance analysis are discussed in Section 4. In Section 5, the conclusions are given.

## 2. System Model

**Figure 1:**
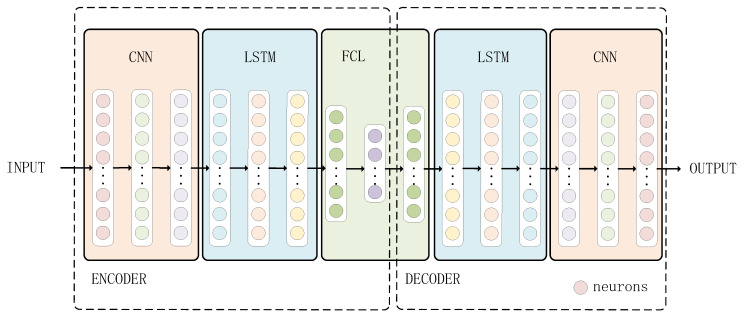
DC-LSTM-AE model architecture.

The overall architecture of the proposed model is illustrated in Figure 1. It is an improved DC-LSTM-AE framework that integrates deep CNN and LSTM within an enhanced autoencoder design. By combining CNN’s capability for extracting spatial features from high-dimensional, complex-structured sensor signals with LSTM’s strength in modeling long-term temporal dependencies, the model jointly learns spatial–temporal representations essential for accurate fault detection. This integration not only improves representation capacity and detection accuracy but also enhances noise robustness and stability in industrial environments, forming the basis for the subsequent data preprocessing, encoder–decoder structure, and detection workflow described below.

In this study, the raw sensor signals are first segmented into fixed-length time windows to capture localized temporal dynamics, and then they are transformed into the frequency domain using the FFT to highlight periodic and harmonic fault-related components. All features are subsequently normalized to a standard scale to eliminate amplitude disparities across sensors and operating conditions. The proposed DC-LSTM-AE model adopts a symmetric encoder–decoder architecture optimized for anomaly detection in high-dimensional industrial time-series data. The encoder consists of a five-layer deep convolutional neural network (CNN), where each layer includes convolution, normalization, non-linear activation, and pooling operations. This hierarchical design enables the progressive extraction of abstract spatial features from frequency-domain representations of raw sensor signals. To mitigate neuron inactivation and improve feature continuity, LeakyReLU is used in place of standard ReLU in the activation layers. The pooling layer is applied to retain certain prominent features maximally, thereby enhancing the model’s fault detection accuracy. Following convolution, an LSTM network is applied to extract temporal features. Subsequently, a fully connected layer (FCL) is used for feature integration and dimensionality reduction. In the decoder, the encoder’s output features are first expanded via an FCL network. Then, the temporal features are restored using an LSTM network. Furthermore, the spatial features of the samples are recovered via a five-layer transposed CNN to ultimately achieve sample reconstruction.

Model training aims to minimize the mean squared reconstruction error between the input and output samples, with an additional L2 regularization term applied to constrain parameter magnitudes and enhance generalization. Once the training loss converges, the model processes the normal training data to compute reconstruction errors, and the detection threshold is determined using the Pauta criterion, defined as the mean reconstruction error plus three standard deviations. During inference, any sample with a reconstruction error exceeding this threshold is classified as faulty, while those below the threshold are considered normal.

The DC-LSTM-AE algorithm model architecture includes an encoder and a decoder. The encoder employs a five-layer CNN for spatial feature extraction, and each layer of the CNN contains a normalization layer, an activation layer, and a pooling layer.

## 3. The Proposed DC-LSTM-AE Algorithms

The working environment of actual industrial production is extremely complex, with complicated and changeable working conditions, and most of the signals collected by sensors are non-stationary and non-linear data [50]. Under these conditions, fault detection and diagnosis require more powerful feature extraction tools to obtain more valuable information from such signals. Deep learning addresses this through powerful feature extraction and learning capabilities enabled by deep neural networks with increased parameters and depth [51].

### 3.1. The CNN Algorithm

CNNs represent a class of deep learning architectures that have been widely adopted for time-series signal analysis. They are particularly effective in processing grid-structured data and have been extensively applied in mechanical equipment fault detection, where they demonstrate superior performance in both signal processing and hierarchical feature extraction [52]. The fundamental principle of CNNs lies in their ability to extract salient local features through convolution operations, while computational complexity is reduced via pooling layers. Core mechanisms such as local connectivity, parameter sharing, and pooling operations collectively enable the network to identify discriminative patterns across diverse signal regions. Local connectivity ensures that each neuron interacts only with a restricted receptive field of the input, in contrast to the fully connected architecture, which links every neuron to all input nodes. Given that localized structures often carry critical diagnostic cues in mechanical fault signals, this property facilitates the targeted extraction of informative patterns from relevant regions, thereby enhancing the model’s fault detection accuracy and robustness. Parameter sharing refers to the use of the same convolution kernel for the connections between all output layer nodes and their corresponding local regions in the input layer. This approach significantly reduces trainable parameter counts, particularly for high-dimensional data, enabling efficient CNN training and deployment in resource-constrained environments. CNN is a hierarchical model whose basic structure consists of an input layer, convolutional layers, pooling layers, fully connected layers, and an output layer with detailed layer specifications provided below [53]:

Input Layer: The input layer serves to receive raw data as input and transmit this information to subsequent layers for feature extraction. In the context of mechanical equipment fault detection, the input data can be time-series data collected by sensors or preprocessed spectrograms.

Convolutional Layers: The convolutional layer extracts local features from the input data, which are leveraged by CNN in mechanical equipment fault diagnosis to identify potential anomalies and faults. The convolutional layer scans the input data using multiple convolution kernels to extract local features in different dimensions. The core formula for the convolution operation is as follows:

(1) $$y\left[i,j,k\right]=\sum_{m=0}^{M-1}\sum_{n=0}^{N-1}x[i+m,j+n]\times w[m,n,k]+b\left[k\right]$$

where *x* is the input signal, *y* is the output signal, *w* is the convolution kernel, *b* is the bias term, and *M* and *N* are parameters representing the size of the convolution kernel.

Pooling Layers: The pooling layer constitutes a critical component of CNN architectures, serving primarily to downsample feature maps and thereby reduce their dimensionality. This operation markedly decreases the number of model parameters while retaining the most informative features, thus improving computational efficiency and enhancing robustness. Pooling also imparts a degree of translation invariance, enabling the network to maintain stable performance under variations in input positioning. In the context of mechanical equipment fault detection, pooling layers facilitate the extraction of key diagnostic patterns from vibration signals or spectrograms, while effectively mitigating the influence of noise.

Fully Connected Layers: The fully connected layer is typically located at the back end of the network. Its main function is to utilize the features extracted by the preceding convolutional and pooling layers for classification, regression, or other tasks. In mechanical equipment fault detection, the fully connected layer transforms analyzed feature representations into diagnostic labels of health states, performing critical fault classification decisions.

### 3.2. The LSTM Algorithm

**Figure 2:**
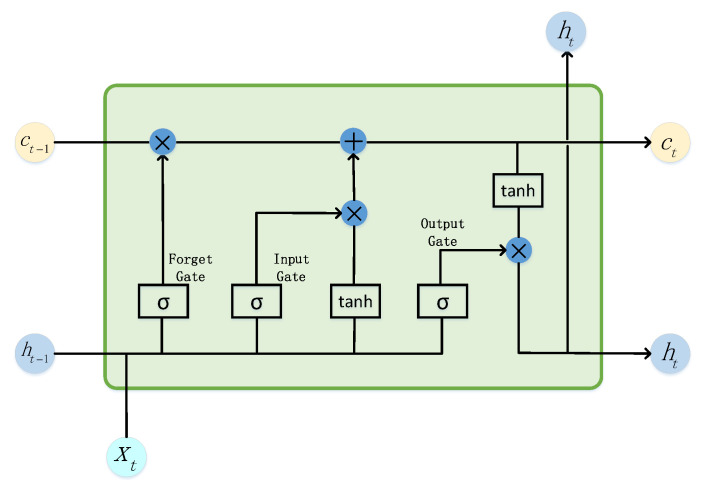
Structural diagram of long short-term memory network.

The LSTM network extends the conventional recurrent neural network (RNN) architecture by introducing memory cells along with input, output, and forget gates. These components enable LSTM to effectively capture long-term dependencies and mitigate the vanishing gradient problem, thereby exhibiting superior performance in modeling long-sequence data [54,55]. The overall architecture of the LSTM network is illustrated in Figure 2.

Input Gate: The input gate computes an affine transformation of the current input $x_{t}$ and the hidden state $h_{t-1}$ output at the previous moment, transforming it via sigmoid activation to the range of [0, 1] to generate a gating signal. Its function is to determine which information is selected for storage in the cell state at the current time step. The specific calculation formula is as follows:

(2) $$i_{t}=sigmoid\left(w_{i}x_{t}+u_{i}h_{t-1}+b_{i}\right)$$

where $w_{i}$ and $u_{i}$ are the weight parameters of the input gate and $b_{i}$ is the bias parameter of the input gate. In mechanical equipment fault detection, the input gate mechanism prevents critical time series information from being discarded, reduces sensitivity to transient irrelevant signals, and consequently extracts key features from long-term data trends.

Forget Gate: The forget gate determines, at each time step, which components of the previous cell state should be retained and which should be discarded through gated operations. This mechanism allows the model to dynamically regulate the information flow within the memory units, thereby adapting to variations in the input sequence. The gating signal is computed as follows:

(3) $$f_{t}=sigmoid\left(w_{f}x_{t}+u_{f}h_{t-1}+b_{f}\right)$$

where $w_{f}$ and $u_{f}$ are the weight parameters of the forget gate and $b_{f}$ is the bias parameter of the forget gate. In fault detection, the forget gate retains the maximum amount of information relevant to fault patterns. Conversely, for random noise or irrelevant operational signals, it filters out such information, effectively suppressing their interference with the judgment of the equipment’s state.

Memory Cell: The memory cell constitutes the core component of the LSTM network, responsible for storing and maintaining long-term dependencies while dynamically regulating the flow of information through its gating mechanisms. Its operation integrates two primary sources of information: the retained state from the previous time step and the newly received input at the current time step. This design enables the model to selectively preserve or update information, thereby mitigating the vanishing gradient problem commonly encountered in conventional RNNs. The corresponding computational formulations are expressed as follows:

(4) $$a_{t}=\tanh\left(w_{a}x_{t}+u_{a}h_{t-1}+b_{a}\right)$$

(5) $$c_{t}=f_{t}c_{t-1}+i_{t}a_{t}$$

where $a_{t}$ denotes the input information and $c_{t}$ is the state information of the memory cell at the current time step. In equipment fault detection, signals collected by sensors may contain a large amount of noise and redundant features. This approach can adaptively filter important information, effectively mitigating key information loss in long time series.

Output Gate: The output gate primarily controls information flow to the next time step. It dynamically selects which historical information to extract from the memory cells for output to the current hidden state. The relevant formula is as follows:

(6) $$o_{t}=sigmoid\left(w_{o}x_{t}+u_{o}h_{t-1}+b_{o}\right)$$

(7) $$h_{t}=o_{t}\cdot \tanh\left(c_{t}\right)$$

where $w_{o}$ and $u_{o}$ are the weight parameters of the output gate, $b_{o}$ is the bias parameter of the output gate, and $o_{t}$ is the gating signal of the output gate with a value range of [0, 1]. In mechanical equipment fault detection, the output gate extracts and outputs content related to fault features from the memory cells. This enables the real-time monitoring of operational status and prediction of future failure risks.

### 3.3. The AE Algorithm

AEs are a class of unsupervised learning models composed of two symmetrical components: an encoder and a decoder [56]. They are widely employed for dimensionality reduction of high-dimensional data by learning compact latent representations. Given a high-dimensional input, the encoder compresses it into a low-dimensional latent space, while the decoder reconstructs the original input from this representation. The network is trained by minimizing the reconstruction error between the input and the reconstructed output, thereby ensuring that the latent space captures the most salient features of the data. In the context of fault detection, anomalies are identified by measuring deviations in reconstruction error between test samples and normal samples [57].

**Figure 3:**
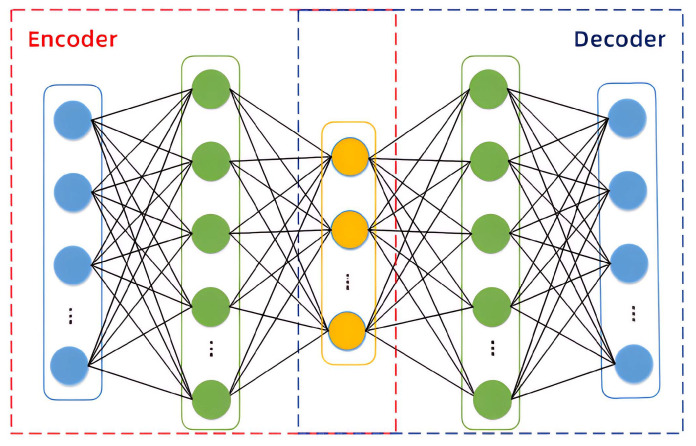
Structure of autoencoder.

The structure of the AE is shown in Figure 3. For high-dimensional input data $X=(x_{1},x_{2},\ldots ,x_{n})$, where *n* represents the number of data points, the corresponding low-dimensional representation is obtained through the encoder. The low-dimensional representation of the data is then input into the decoder to obtain the corresponding output data. Assuming the input data is $x_{i}$, the formulas for the encoding and decoding processes can be expressed as follows:

(8) $$z_{i}=\sigma_{e}\left(w_{e}x_{i}+b_{e}\right)$$

(9) $${\hat{x}}_{i}=\sigma_{d}\left(w_{d}x_{i}+b_{d}\right)$$

where $z_{i}$ is the low-dimensional feature vector obtained by the encoder; $\sigma_{e}$ and $\sigma_{d}$ represent the activation functions of the encoder and decoder, respectively; $w_{e}$ and $b_{e}$ are the weights and biases of the encoder; $w_{d}$ and $b_{d}$ are the weights and biases of the decoder; and $\hat{x}$ denotes the output data obtained by the decoder.

During the training process, the loss function of AE generally selects the MSE loss function:

(10) $$J\left(w,b\right)=\frac{1}{n}\sum_{i=1}^{n}{\left(x_{i}-{\hat{x}}_{i}\right)}^{2}$$

The aforementioned function iteratively updates the network parameters through backpropagation combined with gradient descent, aiming to minimize the reconstruction error between the output and the input data. Upon convergence, the intermediate hidden layer encodes a compact, low-dimensional representation of the original high-dimensional input, effectively capturing its essential features. During inference, if the AE network encounters input data that substantially deviates from the distribution of the training set, its reconstruction performance deteriorates, leading to a pronounced increase in reconstruction error. By quantitatively comparing reconstruction errors across samples, anomalous instances can be distinguished from normal ones with high sensitivity.

### 3.4. The DC-LSTM-AE Algorithm

#### 3.4.1. Model Training

To ensure that the DC-LSTM-AE model effectively captures both spatial and temporal characteristics of vibration signals, the architecture integrates a five-layer convolutional encoder followed by an LSTM-based temporal feature extractor and a decoder for reconstruction. This study substitutes LeakyReLU for the traditional ReLU function in activation layers to mitigate the representational capacity degradation caused by neuronal deactivation, which occurs when ReLU outputs zero for negative inputs. LeakyReLU addresses this issue by introducing a small non-zero gradient (typically 0.01 or 0.02) in the negative domain. Its mathematical formulations are as follows:

(11) $$f\left(x\right)=\left\{\begin{array}{c} x,\;ifx\geq 0\\ \alpha x,\;ifx<0 \end{array}\right.$$

where α is a small constant; this ensures that when the input is negative, the neuron avoids complete deactivation by retaining a small gradient, thereby alleviating vanishing gradients and preserving the network’s learning capacity. The pooling layer is applied to retain certain prominent features maximally, thereby enhancing the model’s fault detection accuracy. Following convolution, an LSTM network is applied to extract temporal features. Subsequently, a fully connected layer (FCL) is used for feature integration and dimensionality reduction.

In the decoder, the encoder’s output features are first expanded via an FCL network. Then, the temporal features are restored using an LSTM network. Furthermore, the spatial features of the samples are recovered via a five-layer transposed CNN to ultimately achieve sample reconstruction. Each layer of the transposed CNN also includes a normalization layer, an activation layer, and an unpooling layer. Here, the LeakyReLU activation function is also used in the activation layer.

With the model architecture established, the training process begins by preparing the input data in a format compatible with both the CNN and LSTM components. This involves segmenting continuous time-domain signals into fixed-length frames through a sliding window approach, ensuring consistency with model input size requirements and FFT preprocessing constraints. The sliding window method segments normal samples into frames. Based on FFT requirements, model input size constraints, and the trade-off between data overlap and information novelty per sample, the window width and step size are set to 2048 and 1024, respectively. This windowing process yields time-series normal samples. Convert the obtained time-series normal signal $S=(s_{1},s_{2},\ldots s_{n})$ into the frequency domain via FFT to generate the training dataset $X=(x_{1},x_{2},\ldots x_{n})$, where *n* denotes the number of normal samples. This can be expressed by the following formula:

(12) $$x_{i,m}=\sum_{k=0}^{l-1}s_{i,k}e^{\frac{-2\pi jmk}{l}}$$

where $x_{i,m}$ represents the *m*-th frequency value of the *i*-th sample, $s_{i,k}$ denotes the *k*-th time-series value of the *i*-th sample, and *l* is the size of the sliding window. Due to the symmetry of the Fourier transform, we retain only half of the spectrum after the Fourier transform to reduce the number of network parameters. The training dataset $X=(x_{1},x_{2},\ldots x_{n})$ is subjected to Z-Score standardization processing [58] to obtain the standardized dataset $X^{\prime }=\left({x^{\prime }}_{1},{x^{\prime }}_{2},\ldots ,{x^{\prime }}_{n}\right)$. The specific formula is as follows:

(13) $${x^{\prime }}_{i,j}=\frac{x_{i,j}-mean\left(X_{j}\right)}{std(X_{j})}$$

where ${x^{\prime }}_{i,j}$ is the standardized value of the *j*-th feature in the *i*-th sample. $x_{i,j}$ is the *j*-th feature value of the *i*-th sample. $X_{j}$ represents the set of all values for the *j*-th feature across all training samples. $mean(X_{j})$ denotes the mean value of the *j*-th feature computed from the training samples. $std(X_{j})$ denotes the standard deviation of the *j*-th feature computed from the training samples. The computed normalization parameters are stored for use during inference.

The preprocessed training dataset $X^{\prime }=\left({x^{\prime }}_{1},{x^{\prime }}_{2},\ldots ,{x^{\prime }}_{n}\right)$ is fed into the designed DC-LSTM-AE model for training, yielding the generated sample set ${\hat{X}}^{\prime }=\left({{\hat{x}}^{\prime }}_{1},{{\hat{x}}^{\prime }}_{2},\ldots ,{{\hat{x}}^{\prime }}_{n}\right)$, the mean squared error between the generated samples, and the input samples is used as the training loss function. In addition, an L2 regularization term is incorporated into the loss function to constrain the model’s complexity and improve its generalization capability. Training is terminated once the loss value converges to a stable level. The specific formula for the loss function is given as follows:

(14) $$loss=\frac{1}{n}\sum_{i=1}^{n}{∥{x^{\prime }}_{i}-{{\hat{x}}^{\prime }}_{i}∥}^{2}+\varepsilon \sum_{p=1}^{p}\omega_{p}^{2}$$

where *P* denotes the total number of parameters in the network, and $\omega_{p}$ represents the p-th parameter of the network. The first term, mean squared error (MSE), quantifies the deviation between the input and its reconstruction, driving the model to progressively approximate the distribution of normal operating conditions and, thus, minimizing reconstruction errors for normal samples. Since the model is trained exclusively on normal data, abnormal samples—which deviate from the learned distribution—produce noticeably larger reconstruction errors, providing a clear discriminative basis for fault detection. The second term, L2 regularization, penalizes the squared magnitude of all network parameters to suppress overfitting, reduce noise sensitivity, and improve adaptability across varying operating environments. The regularization coefficient ε directly influences the trade-off between model complexity and generalization: an excessively large value can lead to underfitting by overly constraining parameters, while an overly small value may retain redundant features and reduce robustness. In this study, ε is determined via cross-validation to achieve an optimal balance between representational capacity and generalization ability. The proposed loss design is computationally efficient and demonstrates strong stability in industrial fault detection tasks, particularly in noisy, multi-condition scenarios.

Upon completion of training, all normal samples are reintroduced to the trained DC-LSTM-AE model to obtain the reconstructed dataset $Y^{\prime }=\left({y^{\prime }}_{1},{y^{\prime }}_{2},\ldots {y^{\prime }}_{n}\right)$, and the reconstruction error for each sample is computed as follows:

(15) $$e_{i}=\sum_{j=1}^{f}{\left({y^{\prime }}_{i,j}-{x^{\prime }}_{i,j}\right)}^{2}$$

where *f* is the number of features. Based on the reconstruction error sequence $E=\left\{e_{1},e_{2},\ldots e_{m}\right\}$ obtained from all normal training samples, it is essential to establish a reasonable and robust decision threshold to enable the trained DC-LSTM-AE model to automatically differentiate between normal and abnormal inputs during inference. The choice of threshold directly impacts the accuracy and stability of the fault detection system: if the threshold is set too low, some normal samples may be misclassified as abnormal (increasing false positives), leading to unnecessary maintenance costs and downtime; conversely, an overly high threshold may cause actual abnormal samples to be overlooked (increasing false negatives), delaying fault diagnosis and potentially resulting in severe equipment failures. Therefore, the threshold determination process must balance high sensitivity with a low false alarm rate while maintaining robustness against the complex and variable noise conditions typically found in industrial environments.

In this study, the Pauta Criterion is adopted for threshold calculation, the core idea of which is based on the statistical properties of the normal distribution. The Pauta Criterion states that in a normal distribution, the vast majority of data (approximately 99.73%) falls within three standard deviations from the mean, and data points outside this range are highly likely to be caused by abnormal factors. Therefore, the threshold is defined as follows:

(16) $$e_{t}=mean\left(E\right)+3\cdot std\left(E\right)$$

where $e_{t}$ denotes the reconstruction error threshold; mean(E) and $std\left(E\right)$ denote the mean and standard deviation of the reconstruction errors for all normal training samples, respectively. This setting implies that if the reconstruction error of a test sample exceeds this range, its deviation from the normal-state distribution surpasses the statistically rare fluctuation interval—there is high confidence that the operating state corresponding to the sample is abnormal.

#### 3.4.2. Anomaly Detection Process

**Figure 4:**
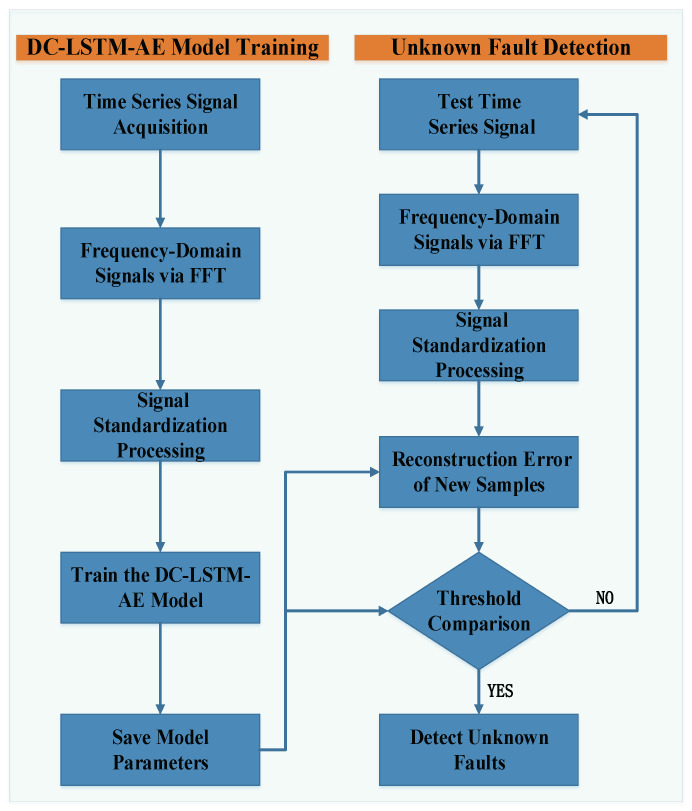
Mechanical equipment unknown fault detection process based on normal samples.

The specific workflow of the proposed mechanical equipment unknown fault detection method, based exclusively on normal samples, is illustrated in Figure 4. As shown in the flowchart, raw vibration signals collected from the equipment’s sensors serve as the primary input for the diagnostic process. These signals are initially examined to reveal potential variations indicative of abnormal operating conditions.

Following data acquisition, the trained DC-LSTM-AE model and the reconstruction error threshold—both determined during the model training phase using the standardized dataset $\hat{X}$—are retrieved from storage. The detection stage then proceeds as follows. The newly acquired time-domain vibration signals are first transformed into the frequency domain using the FFT, as described in (12), enabling the capture of periodic characteristics and frequency-related fault signatures that may not be evident in the time domain alone. To maintain statistical consistency with the training data, the resulting frequency-domain features are standardized using the mean and standard deviation parameters obtained during the Z-score normalization of the normal-sample training set.

The standardized frequency-domain samples are subsequently fed into the preserved DC-LSTM-AE model, which reconstructs the input signals based on learned normal patterns. For each test sample, the reconstruction error is computed as the squared difference between the original and reconstructed features, aggregated across all dimensions. This error is then compared against the precomputed threshold $e_{t}$, defined using the Pauta Criterion to balance sensitivity and specificity in the classification decision.

If the reconstruction error does not exceed $e_{t}$, the operational state of the equipment is classified as normal. Conversely, when the reconstruction error surpasses this threshold, the sample is labeled as abnormal, triggering an automated alarm. This alarm acts as an immediate alert to on-site maintenance personnel, prompting timely inspection and corrective measures to prevent escalation of faults, reduce production downtime, and minimize the risk of safety incidents. The reliance on a statistically grounded threshold, combined with the end-to-end learned reconstruction capability of the DC-LSTM-AE, ensures that the system can detect unknown faults robustly, even in the absence of fault-specific training data, thereby enhancing its applicability in complex and variable industrial environments.

## 4. Results Simulation and Discussion

To fully validate the practicality and effectiveness of the proposed normal-sample-based fault detection method for mechanical equipment, multiple datasets were selected for experimental verification, including the gearbox dataset from Southeast University and the constant-speed water pump dataset collected in actual factories. Furthermore, the proposed method was extensively compared with established anomaly detection techniques and various autoencoder network architectures. These comparisons aimed to demonstrate the method’s superiority in detecting unknown faults.

### 4.1. Experimental Setup

Without loss of generality, the simulation parameters were set as follows in Python 3.9 for WIN10. The computer configuration was as follows: the processor (CPU) was an Intel(R) Core(TM) i7-10750H CPU @ 2.60 GHz; the clock speed was 2.59 GHz; the memory (RAM) was 1024 GB with an NVIDIA GeForce GTX 1650 Ti. After the data collected from the sensor undergoes sliding window sampling and FFT processing, each sample has a dimensionality of 1024. Table 2 details the DC-LSTM-AE network architecture and layer output dimensions, designed according to this input size. For model training, we used the Adam optimizer with a learning rate of 0.001, training for 500 epochs with a batch size of 128.

### 4.2. Experimental Datasets

#### 4.2.1. The Gearbox Dataset from Southeast University

**Figure 5:**
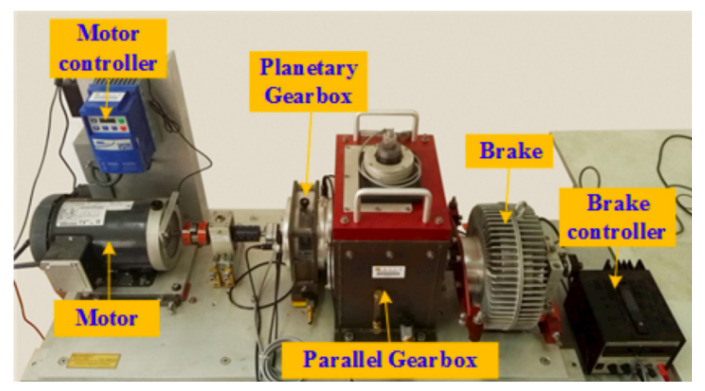
Gearbox test rig of Southeast University dataset.

In this study, the gearbox dataset from Southeast University was collected using a dedicated gearbox test rig, as illustrated in Figure 5. The test rig comprises a motor, motor controller, planetary gearbox, reduction gearbox, brake, and brake controller. Data acquisition was conducted under a controlled experimental design, in which the rotational speed was fixed at 20 Hz and the load was maintained at 0 V to ensure consistency across all measurements. Both healthy operating conditions and four distinct fault types were recorded, namely gear crack, tooth breakage, root crack, and surface wear.

During data preparation, vibration signals from multiple sensors were initially collected; however, to maintain consistency and reduce signal variability, only the x-direction vibration signals of the planetary gearbox were selected as the primary input source. The raw signals underwent preprocessing steps including de-noising, normalization, and segmentation into fixed-length time windows, ensuring that both normal and fault samples were uniformly represented. The dataset was then partitioned into training and testing subsets in an 8:2 ratio, with only normal samples used for model training to align with the one-class anomaly detection setting.

To demonstrate the proposed algorithm’s ability in mechanical equipment fault detection without relying on fault samples, the normal samples were first randomly divided into training and test sets at an 8:2 ratio. Subsequently, 200 samples were randomly selected from each type of fault sample as test samples for unknown faults. The model was trained exclusively on normal samples, while testing utilized both normal samples and samples from four fault types. The detailed division of the number of training and test samples in the dataset is shown in Table 3.

#### 4.2.2. Constant-Speed Water Pump Dataset

To evaluate the practical applicability of the proposed model, a constant-speed water pump dataset collected from an operational industrial facility was employed for experimental validation. In addition to normal operational measurements, this dataset includes bearing fault condition data. Consistent with the experimental protocol, only normal samples were utilized for model training, whereas both normal and fault samples were included in the testing phase. For each dataset, 80% of the normal samples were used for training and 20% for testing, with abnormal samples reserved exclusively for the test set. The specific division of the number of training and test samples is shown in Table 4.

### 4.3. Evaluation Metrics

Due to a significant imbalance between fault and normal data proportions, mechanical equipment anomaly detection faces substantial class imbalance challenges. To ensure that this imbalance does not bias the evaluation, we selected four complementary performance metrics—Accuracy (ACC), Precision (PRE), Recall (REC), and F1-score. While ACC provides an overall measure of classification correctness, it may be inflated under imbalanced conditions; therefore, PRE and REC were included to capture, respectively, the model’s ability to minimize false positives and false negatives. The F1-score, as the harmonic mean of PRE and REC, offers a balanced assessment when the two are in tension. This combination of metrics provides a more robust and fair evaluation than ACC alone, implicitly mitigating the bias introduced by class imbalance. In addition, all comparative models were trained and tested under the same 8:2 split and evaluation criteria, ensuring that the reported differences in performance are attributable to model capability rather than dataset distribution. The closer the values of these indicators are to 1, the better the detection effect. The specific calculation formulas for each indicator are as follows:

(17) $$ACC=\frac{TP+TN}{TP+TN+FN+FP}$$

(18) $$PRE=\frac{TP}{TP+FP}$$

(19) $$REC=\frac{TP}{TP+FN}$$

(20) $$F1=\frac{2*PRE*REC}{PRE+REC}$$

Herein, TP denotes the number of actual fault samples correctly predicted as faulty, TN represents the number of actual normal samples correctly predicted as normal, FN signifies the number of actual fault samples incorrectly predicted as normal, and FP indicates the number of actual normal samples incorrectly predicted as faulty. The $F_{1}$-score, defined as the harmonic mean of precision (PRE) and recall (REC), provides a comprehensive evaluation of fault detection performance by integrating these complementary metrics.

To enhance the transparency and reproducibility of the experiments, all evaluation steps in this study followed a clearly defined procedure. The raw vibration data from each dataset were first segmented using a sliding window and transformed into the frequency domain via FFT. Standardization was performed using Z-score normalization, with statistics computed from the training set only. The datasets were split into training and testing sets with an 8:2 ratio, ensuring that only normal samples were used for model training to simulate real-world unknown fault scenarios. Hyperparameters, including learning rate, batch size, and number of epochs, were selected through preliminary validation experiments, while random seeds were fixed to maintain result reproducibility. All models, including the proposed DC-LSTM-AE and comparison baselines (AE, DCAE, iForest, OCSVM, and Transformer), were implemented under the same computational environment to ensure fairness in comparison, and all comparative models were evaluated without any modifications to their original architectures.

### 4.4. Experimental Results and Analysis

To evaluate the abnormal sample detection performance of the proposed model, we benchmarked it against two common anomaly detection algorithms: Isolation Forest (iForest) and One-Class Support Vector Machine (OCSVM). In the iForest algorithm, the number of isolation trees was set to 100, the maximum depth of each tree was 8, and each tree randomly selected 256 samples for training. For the OCSVM algorithm, the Gaussian kernel function served as the kernel, and the proportion of abnormal samples was set to 0.02. Furthermore, to evaluate the superiority of the designed DC-LSTM-AE model in feature extraction compared to traditional autoencoder networks, comparisons were also conducted with the traditional autoencoder (AE) and the deep convolutional autoencoder (DCAE) [46,59]. The network depth of both AE and DCAE was the same as that of the DC-LSTM-AE model. In the AE model, all hidden layers were composed of fully connected layers, while in the DCAE network, the hidden layers consisted of convolutional layers and fully connected layers. In addition, a standard Transformer model was included in the comparative experiments to assess its applicability in mechanical equipment fault detection. The Transformer was configured with a multi-head self-attention mechanism, positional encoding, and feed-forward layers following the original architecture. This model leverages its strong capability in modeling long-range dependencies in time-series signals, providing a valuable benchmark for evaluating the benefits of combining convolutional spatial feature extraction with temporal modeling in the proposed DC-LSTM-AE. During the experiment, each algorithm was trained using only normal samples and tested using both normal and fault samples, with the ratio of normal samples for training to testing set at 8:2.

#### 4.4.1. Experimental Verification of Southeast University Gearbox Dataset

**Figure 6:**
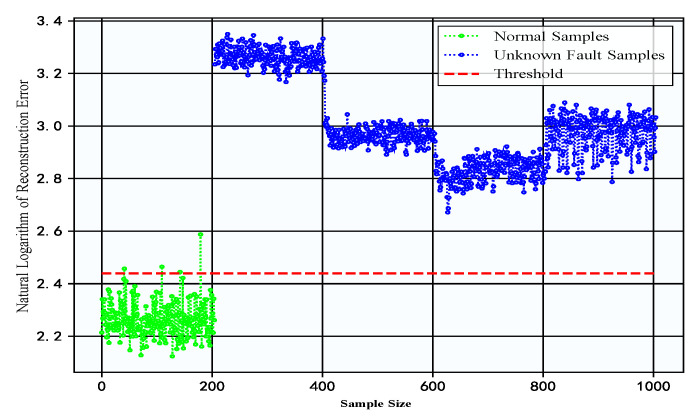
Reconstruction error of test data for the DC-LSTM-AE model in the gearbox dataset.

To validate the effectiveness of the proposed normal-sample-based mechanical fault detection method, we first conducted experiments using the Southeast University (SEU) gearbox dataset. Given the scarcity of fault samples in industrial settings, our method’s practicality was demonstrated by training exclusively on normal samples while evaluating performance using both normal and fault samples. Figure 6 presents the DC-LSTM-AE model’s reconstruction errors for test data. The results demonstrate lower reconstruction errors for normal samples compared to fault samples. Moreover, the reconstruction errors of normal test samples generally remain below the threshold, whereas those of unknown fault samples mostly exceed it. The unknown fault detection successfully identifies all four fault types while maintaining high classification accuracy for normal samples. Furthermore, distinct characteristics of the four fault types result in varying degrees of deviation from the normal sample. Since the model is only trained on normal samples, the differences in reconstruction errors between different types of faults are relatively large, but show little variation within the same fault type, because samples of the same fault share similar characteristics. This is also reflected in Figure 6.

**Figure 7:**
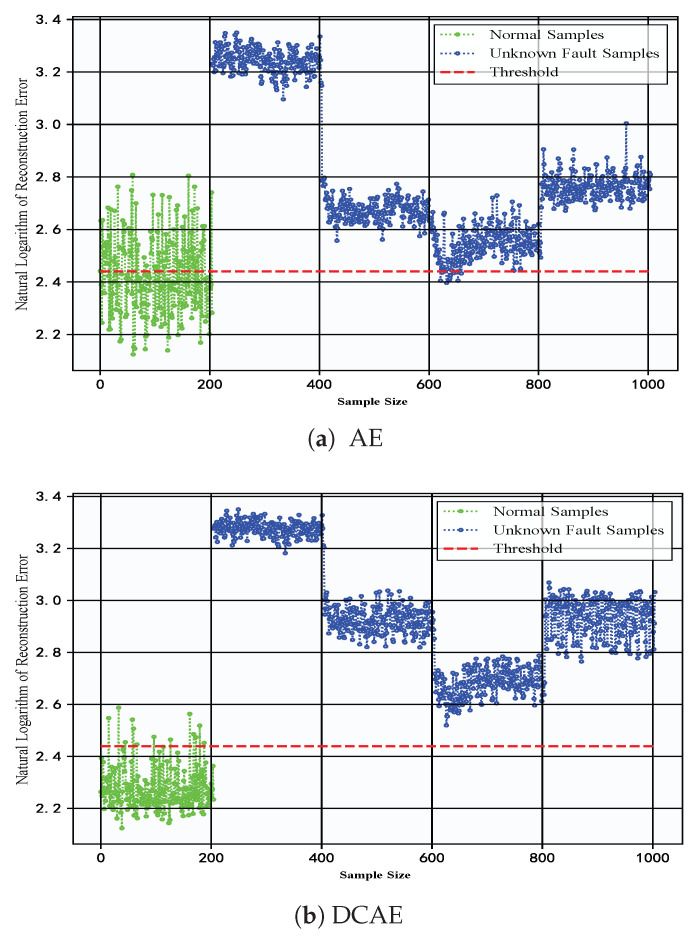
Reconstruction errors of test data for the AE model and DCAE model in the gearbox dataset.

The proposed DC-LSTM-AE framework integrates a five-layer CNN with LSTM modules within the encoder of a traditional autoencoder. By combining CNN-based spatial feature extraction with LSTM-based temporal modeling, the model effectively learns spatiotemporal representations, thereby enhancing its ability to detect previously unseen faults. To evaluate the detection advantages of the proposed DC-LSTM-AE model for unknown fault samples, the AE model and the DCAE model were selected for comparative analysis. As shown in Figure 7, the reconstruction errors of the AE and DCAE models on the gearbox dataset reveal clear performance differences. The AE model, due to its limited feature extraction capability, misclassifies most unknown fault samples as normal when their characteristics resemble those of normal samples, despite correctly identifying certain normal samples and specific fault types. In contrast, the incorporation of CNN in the DCAE model substantially improves feature extraction capability, enabling a more accurate detection of unknown faults. However, the absence of temporal feature modeling limits its performance, resulting in a relatively small separation between the reconstruction errors of unknown fault samples and those of normal samples. Consequently, when the fault features are highly similar to those of normal samples, the DCAE model still exhibits a risk of detection errors. However, for the DC-LSTM-AE model, due to the large difference in reconstruction errors between normal samples and fault samples, it exhibits superior detection capability for unknown faults and precisely identifies even small equipment faults.

**Figure 8:**
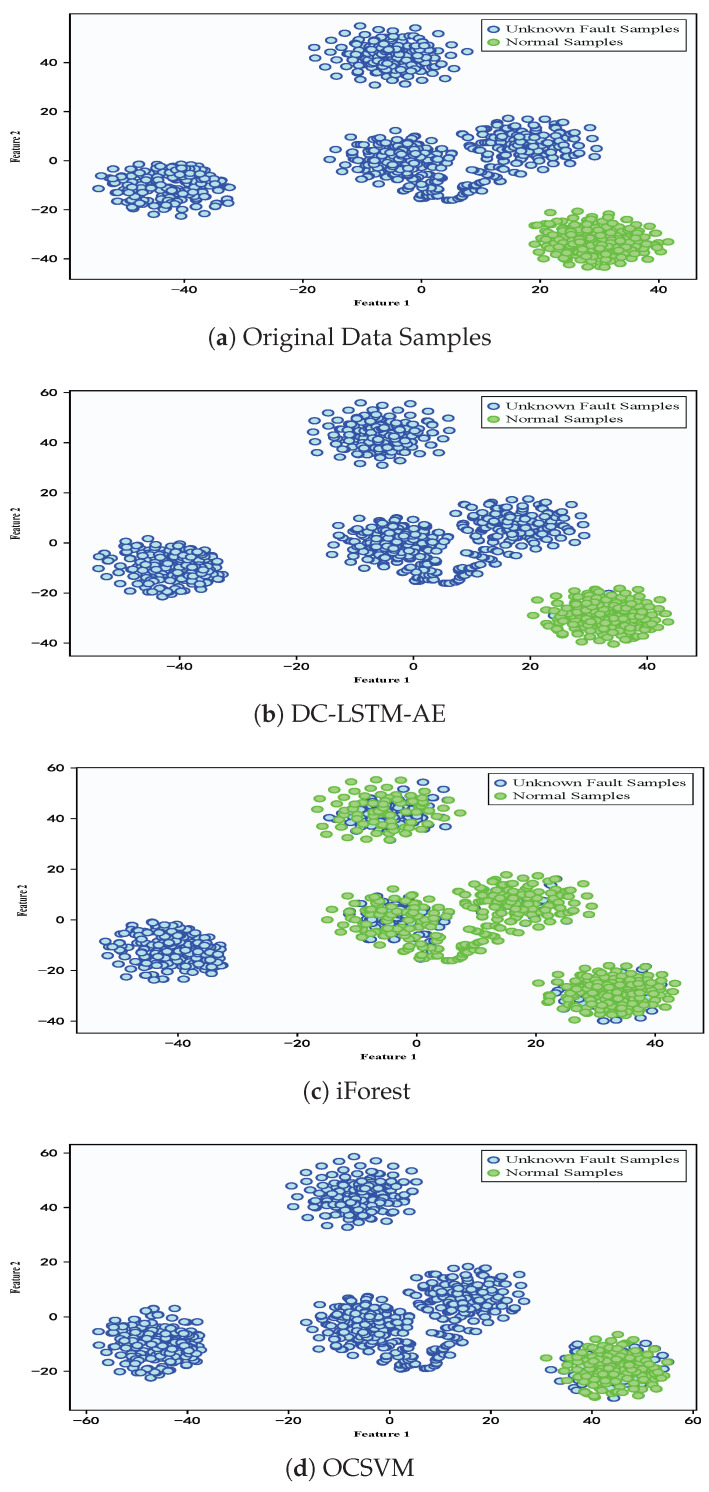
Detection results of different algorithms for test samples in the gearbox dataset.

To demonstrate the advantages of the proposed DC-LSTM-AE model for mechanical equipment unknown fault detection, the iForest model and the OCSVM model were selected for comparative analysis. The T-SNE algorithm was used to visualize the high-dimensional features of the test samples [60], and Figure 8 shows the detection results of different algorithms for the test samples of the gearbox dataset. The first image shows the distribution of normal and fault samples in the real test samples. The detection results of the DC-LSTM-AE model show that all unknown fault samples are correctly detected, and only a few normal samples are mistakenly detected as unknown fault samples. However, although the traditional iForest algorithm accurately detects most normal samples, most unknown fault samples are mistakenly detected as normal samples. The OCSVM algorithm correctly detects all unknown fault samples, but most normal samples are detected as fault samples, which will lead to many false alarms in practical applications. In comparison, the Transformer-based model achieves performance superior to AE, DCAE, iForest, and OCSVM but still falls short of the proposed DC-LSTM-AE method. While its self-attention mechanism effectively captures global dependencies in the data, enhancing its ability to detect unknown faults, the lack of explicit convolutional spatial feature extraction and LSTM temporal modeling results in slightly lower accuracy and F1-score than DC-LSTM-AE. Nevertheless, the Transformer remains a competitive baseline, particularly in scenarios with rich and diverse training data. Similarly, the Ensemble Auto-Encoder (EAE) demonstrates strong unsupervised feature learning capabilities by aggregating multiple autoencoders to enhance robustness against noise and local optima. In the gearbox dataset, EAE outperforms conventional AE and OCSVM, achieving accuracy and F1-scores comparable to DCAE. However, it still underperforms relative to DC-LSTM-AE, primarily due to its limited ability to explicitly model temporal dependencies in sequential signals.

**Figure 9:**
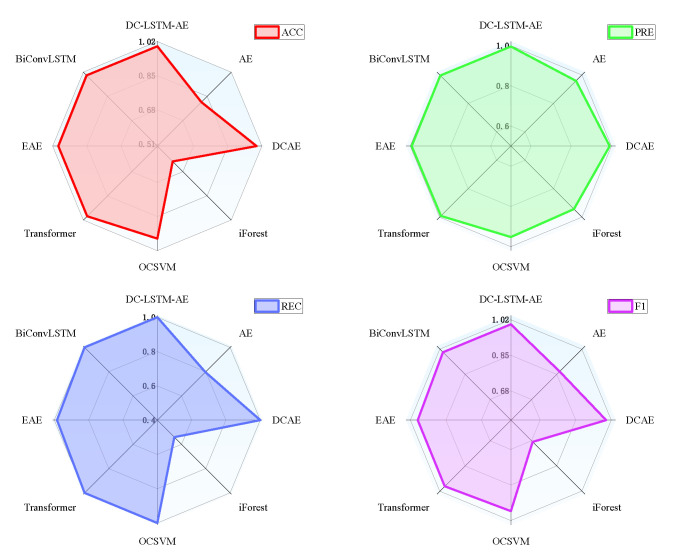
Unknown fault detection performance of various algorithms on gearbox dataset.

In contrast, the BiConvLSTM model leverages bidirectional convolutional layers and recurrent temporal modeling to jointly capture spatial and temporal fault features. This dual representation enables BiConvLSTM to achieve performance approaching that of DC-LSTM-AE, particularly in recall and F1-score, confirming its effectiveness for complex rotating machinery diagnostics. Nonetheless, despite its strong results, the proposed DC-LSTM-AE maintains a consistent advantage across all metrics, validating its superior ability to balance spatiotemporal representation and anomaly detection performance. For the gearbox dataset, the detailed experimental results of each algorithm are shown in Figure 9. It can be seen from the table that the proposed DC-LSTM-AE-based method for mechanical equipment unknown fault detection outperforms all comparable algorithms across all evaluation metrics when trained exclusively on normal samples.

#### 4.4.2. Experimental Validation of Constant-Speed Water Pump Dataset

**Figure 10:**
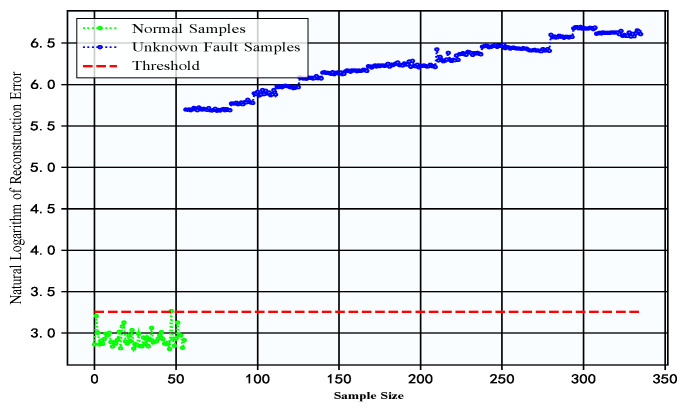
Reconstruction errors of test data for DC-LSTM-AE model in constant-speed water pump dataset.

To further validate the effectiveness of the normal-sample-based mechanical equipment unknown fault detection in practical industrial environments, experimental validation was conducted on a constant-speed water pump dataset collected from actual factories. For this dataset, only normal samples were used for model training, and both normal and unknown fault samples were used for testing, with the training-to-testing ratio of normal samples set at 8:2. Figure 10 shows the reconstruction errors of the test data from the DC-LSTM-AE model for the constant-speed water pump dataset. It can be seen that a significant difference exists in reconstruction errors between normal samples and unknown fault samples. According to the detection criteria for unknown faults, only one normal sample in the test set was misclassified as faulty, while all unknown fault samples were correctly identified. This result demonstrates that the proposed method achieves excellent detection performance for unknown faults in practical industrial environments. Furthermore, an analysis of the reconstruction errors for unknown fault samples shows a progressive increase in error magnitude over time, indicating that the severity of equipment degradation intensifies as faults develop. Owing to its high sensitivity to early fault patterns, the proposed method can detect anomalies at the initial stage of fault inception. Consequently, the timely issuance of early warnings and the implementation of preventive maintenance during the early fault stage can effectively mitigate further equipment deterioration, enhance production efficiency, protect the safety and property of frontline workers, and reduce the likelihood of serious production accidents.

**Figure 11:**
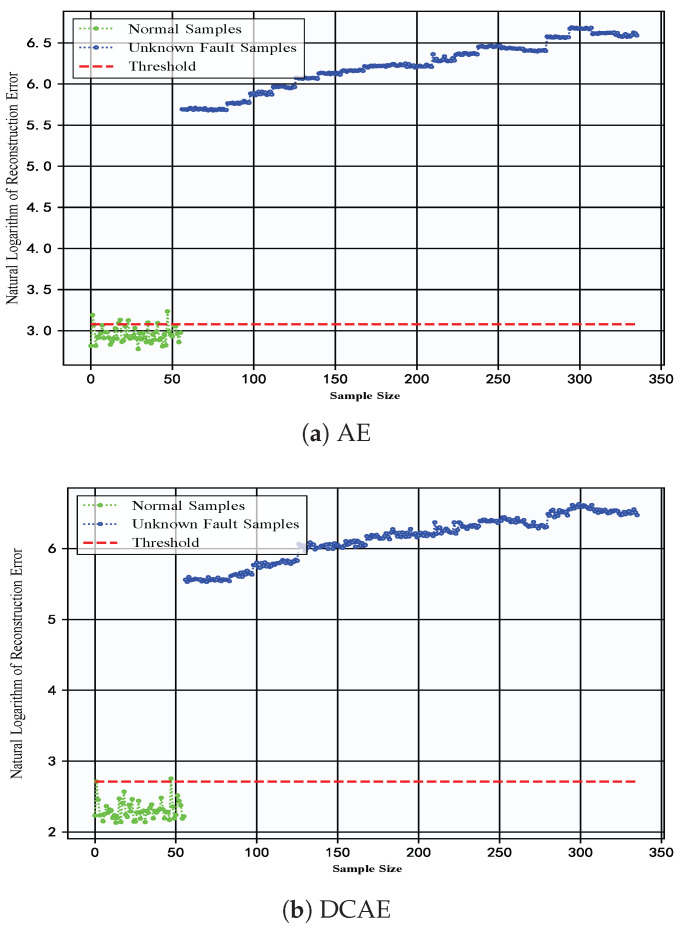
Reconstruction errors of test data for AE model and DCAE model in constant-speed water pump dataset.

To highlight the superiority of the DC-LSTM-AE model over traditional autoencoder structures in detecting unknown faults, the AE and DCAE models were also tested on the constant-speed water pump dataset. Figure 11 presents the reconstruction errors of the AE and DCAE models on the test data from this dataset. A pronounced gap can be observed between the reconstruction errors of normal samples and those of unknown fault samples, which can be attributed to substantial feature dissimilarity. However, the limited feature extraction capability of the AE model results in suboptimal reconstruction of normal samples, causing certain normal samples to be misclassified as faulty. In contrast, the proposed DC-LSTM-AE model leverages the complementary strengths of CNN-based spatial feature extraction and LSTM-based temporal modeling, thereby enabling more accurate reconstruction and effective differentiation between normal and unknown fault samples. Compared to the AE and DCAE models, the DC-LSTM-AE model achieves greater separation between normal and unknown fault reconstruction errors while maintaining more stable error distributions. These results validate the superiority of the DC-LSTM-AE model over traditional autoencoder structures in detecting unknown faults.

**Figure 12:**
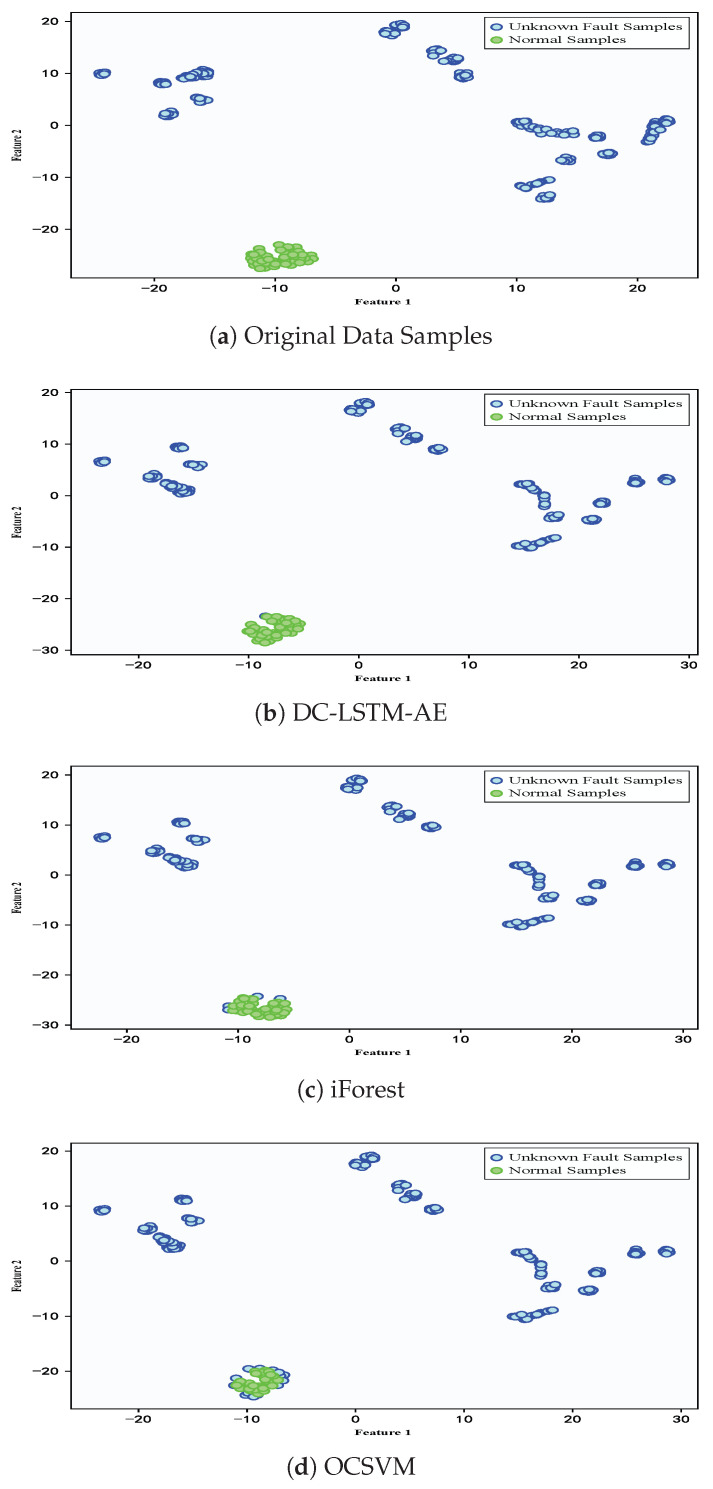
Detection results of different algorithms on the test samples of the constant-speed water pump dataset.

**Figure 13:**
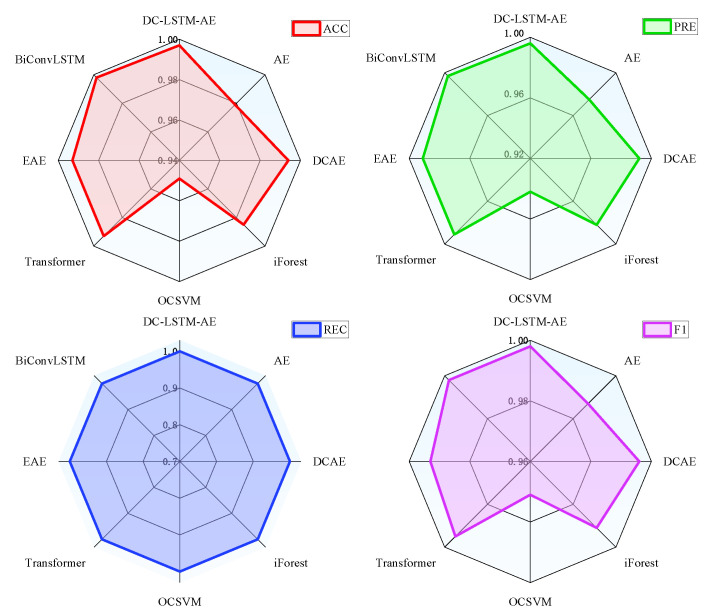
Unknown fault detection performance of various algorithms on constant-speed water pump dataset.

To demonstrate the advantages of the normal-sample-based mechanical equipment unknown fault detection over traditional machine learning algorithms in practical industrial environments, experiments were conducted on the iForest and OCSVM algorithms using the constant-speed water pump dataset collected from actual factories. Figure 12 shows the detection results of different algorithms for the test samples of the constant-speed water pump dataset. First, significant feature dissimilarity between normal and unknown fault samples enables the model to readily distinguish unknown fault samples when trained on normal samples. The Transformer model achieves competitive detection performance due to its global attention mechanism. However, compared to DC-LSTM-AE, it exhibits slightly lower accuracy and precision in identifying normal samples. The Ensemble Auto-Encoder (EAE) further improves over conventional AE through ensemble learning, but its lack of temporal modeling restricts performance gains. In contrast, BiConvLSTM leverages both convolutional and recurrent structures, yielding a higher recall and F1-score, though still marginally inferior to DC-LSTM-AE in precision. However, in terms of detecting normal samples, both the iForest and OCSVM algorithms misclassified some normal samples as unknown fault samples. The DC-LSTM-AE model, benefiting from the effective extraction of spatiotemporal information of samples, has a strong reconstruction capability and high recognition accuracy for normal samples, resulting in a higher detection accuracy for normal samples. Moreover, Figure 13 shows the detailed detection results of various algorithms in the experiment on the constant-speed water pump dataset. It can be seen from the table that the proposed DC-LSTM-AE-based method for mechanical equipment unknown fault detection algorithm based on the DC-LSTM-AE model outperforms other algorithms in all metrics when only normal samples are used for training.

## 5. Conclusions

This paper presents a novel mechanical equipment unknown fault detection that addresses the challenge of limited fault samples by training solely on normal samples, enabling accurate detection of unknown faults without relying on fault samples. To overcome the insufficient expressiveness of conventional autoencoders for high-dimensional nonlinear signals, the proposed DC-LSTM-AE model incorporates both CNN and LSTM modules to jointly extract spatial and temporal features, thereby improving its ability to detect unknown faults. To further improve training stability and model generalization, the input signals are transformed into the frequency domain using FFT, and an L2 regularization term is introduced with dynamic adjustment via cross-validation. These enhancements make the model well-suited for real-time deployment in resource-constrained industrial environments. The structure of the DC-LSTM-AE model is described in detail, and a mechanical equipment unknown fault detection based on normal samples is designed based on this model, with a detailed elaboration of the detection process for unknown fault samples. To validate the effectiveness of this method, the gearbox dataset from Southeast University and the constant-speed water pump dataset collected from actual factories were selected for experimental validation. Comparative experiments were conducted with traditional autoencoder models and common unknown fault detection algorithms. The experimental results showed that the proposed method outperformed other algorithms in multiple metrics for unknown fault detection when only trained on normal samples.

Owing to its strong feature extraction capability and generalizable architecture, the model exhibits high sensitivity to unknown faults and is not limited to specific equipment types. As long as sufficient normal-state time-series data are available, the method can be extended to other industrial machinery, such as motors, wind turbines, and compressors. The average inference time per sample was measured at 2.37 ms, ensuring real-time feasibility for industrial monitoring systems. Additionally, peak memory usage during inference was approximately 512 MB, demonstrating the model’s efficiency and suitability for deployment in resource-constrained industrial devices. Future research will focus on incorporating multidimensional data fusion techniques into the autoencoder framework. By integrating heterogeneous sensor modalities—such as vibration, temperature, and acoustic signals—the model’s perception and diagnostic capabilities can be further enhanced. This multidimensional fusion approach is expected to improve the recognition of abnormal patterns and unknown fault types, laying the foundation for a more robust and generalized deep-learning-based fault detection system in industrial environments.

## Figures and Tables

**Table 1:** Comparison of DC-LSTM-AE and related methods.

Method	Architecture	Fault Detection Approach	Capability for Unknown Faults
AE	Fully connected	Reconstruction error	Low faults
LSTM	Recurrent layers	Classification-based	Low
CNN-LSTM	CNN + LSTM	Classification-based	Low
DC-LSTM-AE (This work)	5-layer CNN + LSTM + AE	Reconstruction error	High

**Table 2:** Network architecture of the DC-LSTM-AE model.

Encoder		Decoder	
Network Layer	Input Dimension	Network Layer	Output Dimension
Input Layer	128×1×1024	FC Layer 3	128×128
Conv Layer 1	128×16×1010	FC Layer 4	128×960
Conv Layer 2	128×32×504	LSTM Network	128×15×256
Conv Layer 3	128×64×251	Deconv Layer 1	128×128×62
Conv Layer 4	128×128×62	Deconv Layer 2	128×64×251
Conv Layer 5	128×256×15	Deconv Layer 3	128×32×504
LSTM Network	128×15×128	Deconv Layer 4	128×16×1010
FC Layer 1	128×128	Deconv Layer 5	128×1×1024
FC Layer 2	128×64	Output Layer	128×1024

**Table 3:** Dataset division for fault detection experiment.

Fault Type	All Samples	Training	Testing
Normal	1022	817	205
Crack on gear	200	0	200
Broken tooth on gear	200	0	200
Crack at gear root	200	0	200
Wear on gear surface	200	0	200

**Table 4:** Dataset division for constant-speed water pump experiment.

Fault Type	All Samples	Training	Testing
Normal	280	224	56
Bearing Fault	280	0	280

## Data Availability

For access to the data used in this study, please contact the corresponding author if needed.
